# Up-Regulation of Superoxide Dismutase 2 in 3D Spheroid Formation Promotes Therapeutic Potency of Human Umbilical Cord Blood-Derived Mesenchymal Stem Cells

**DOI:** 10.3390/antiox9010066

**Published:** 2020-01-11

**Authors:** Miyoung Lee, Bo Ram Song, Dong Hyun Kim, Jueun Ha, Minju Lee, Soo Jin Choi, Wonil Oh, Soyoun Um, Hye Jin Jin

**Affiliations:** Biomedical Research Institute, MEDIPOST Co., Ltd., Seongnam 13494, Korea; hayov@medi-post.co.kr (M.L.); brsong@medi-post.co.kr (B.R.S.); pooh1994@medi-post.co.kr (D.H.K.); 522win@medi-post.co.kr (J.H.); lmj262@medi-post.co.kr (M.L.); sjchoi@medi-post.co.kr (S.J.C.); wioh@medi-post.co.kr (W.O.)

**Keywords:** superoxide dismutase 2, spheroid, mesenchymal stem cell, cell therapy

## Abstract

Umbilical cord blood-derived mesenchymal stem cells (UCB-MSCs) are accessible, available in abundance, and have been shown to be a promising source that can regenerate cartilage in patients with osteoarthritis or other orthopedic diseases. Recently, a three-dimensional (3D) cell culture system was developed to mimic the naive tissue microenvironment. However, the efficacy of cells generated from the 3D spheroid culture system has not yet been elucidated. In the present study, we demonstrate the changes in superoxide dismutase 2 (SOD2) gene expression, an indicator of oxidative stress, on 3D spheroid MSCs. Moreover, siRNA transfection and neutralizing antibody investigations were performed to confirm the function of SOD2 and E-cadherin. Overall, we found that SOD2 siRNA transfection in the spheroid form of MSCs increases the expression of apoptotic genes and decreases the clearance of mitochondrial reactive oxygen species (ROS). As a result, we confirm that 3D spheroid formation increases E-cadherin and SOD2 expression, ultimately regulating the phosphoinositide 3-kinase (PI3K/pAkt/pNrf2 and pERK/pNrf2 signaling pathway. Additionally, we show that SOD2 expression on 3D spheroid MSCs affects the regeneration rates of destructive cartilage in an osteoarthritic model. We postulate that the impact of SOD2 expression on 3D spheroid MSCs reduces oxidative stress and apoptosis, and also promotes cartilage regeneration.

## 1. Introduction

Mesenchymal stems cell (MSCs), which are multipotent progenitor cells, can be isolated from adult bone marrow or other prenatal tissues. These promising cell sources have been previously shown to regenerate several tissues types, such as neurons, bone, and cartilage, regardless of origin [1,2]. Further, MSCs have been shown to have the capacity to repair cartilage damage in osteoarthritic and other orthopedic models [3,4,5]. Osteoarthritis is the main cause of joint pain and stiffness that results from the breakdown of cartilage tissue and underlying bone in joints. In severe cases, surgeries such as joint replacement or osteotomy are necessary; however, they incur high costs [6]. The symptoms, namely joint swelling and difficulty of movement, develop over many years [7,8]. Umbilical cord blood-derived MSCs (UCB-MSCs) are easily accessible, available in abundance, and have been shown to be a promising source for the potential clinical applications of stem cell therapy, especially osteoarthritis [9].

The current standard of cell culture systems involves the plating of cells in a monolayer. However, two-dimensional (2D) culture systems lack the ability to mimic the naive tissue microenvironment. Specifically, chondrocytes lose their native phenotype of expressing type I collagen; however, this does not occur in articular cartilage formed in a monolayer culture [10]. Furthermore, common cell culture systems for chondrogenesis are the pellet culture and micromass culture systems [11,12]. Three-dimensional (3D) cell culture increases direct cell-to-cell interaction, starting from an initial aggregation that is caused by the tight interactions of long extracellular matrix fibers with multiple arginylglycylaspartic acid (RGD) motifs with integrin on the surface of cell membranes. As a result, these interactions elicit the enhanced expression of N-cadherin in the cell membrane [13]. The 3D cell aggregate (also known as spheroid) culture system is a new approach to increase cell-cell interactions. 3D spheroid culture comprises the complexity of tissues and mimics physiological conditions like cell-cell and cell-matrix interactions [14]. MSCs in spheroid culture showed higher levels of gene expression in pluripotency marker genes, *NANOG*, SRY-Box transcription factor 2 (*SOX-2*), and octamer binding transcription factor 4 (*OCT4*), with the enhanced capacity of colony formation, suggesting an increase in stemness [15,16]. Moreover, 3D spheroid culture improved the survival of stem cells after transplantation by upregulating the anti-apoptotic factor, Bcl-2, and downregulating other apoptotic factors such as Bcl-2-associated X protein (BAX) [17,18]. Additionally, 3D spheroid formation enhanced the therapeutic potential of spheroid MSCs as increases have been observed in their angiogenic, anti-inflammatory, and immunomodulatory properties [16,17,19,20,21].

Recently, the application of aggregated adipose-derived stem cells (ACSC), bone marrow-derived stem cells (BMSCs), and Wharton’s jelly cells (WJCs) from umbilical cord demonstrated increased osteogenic and chondrogenic activity in osteochondral disease models [22,23,24,25,26]. Additionally, preclinical animal studies, whereby animals received transplantation of 3D spheroid UCB-MSCs in cases of liver disease, have reported increases of hepatic differentiation. This highlights the improved liver regenerative capabilities of the UCB-MSCs [27,28]. Transplantation of UCB-MSCs enhanced endothelial and cardiomyocyte differentiation in a rat myocardial infarction model [29]. Further, the transplantation of these UCB-MSCs has shown to enhance vascularization in models of ischemia [17,30]. However, a 3D spheroid culture of UCB-MSCs has not yet been fully elucidated in the context of osteochondral disease. Furthermore, there is no evidence on a key factor for 3D spheroid MSCs which will enable their survival upon transplantation.

Mitochondrial superoxide dismutase 2 (SOD2) is a well-known anti-apoptotic molecule scavenging mitochondrial reactive oxygen species (ROS). As a result, SOD2 has roles in protection against cell death, oxidative stress, ionizing radiation, and inflammatory cytokines [31,32]. Mitochondrial SOD2 regulates epithelial-mesenchymal transition and, as a result, the downregulation of E-cadherin, an epithelial marker, in SOD2 knockdown cells is partly inhibited [33]. Additionally, an E-cadherin increase in spheroid formation is proposed to be a key factor that elicits the formation of the spheroid and promotes therapeutic potency of the proliferative and paracrine activity seen in UCB-derived MSCs [29].

In this study, we propose that our 3D spheroid MSC culture system, containing aggregated cells tightly adhering to each other, can maintain intracellular functions that are similar to physiological conditions in vivo. 3D spheroid formation of MSCs transfected with siRNA SOD2 increased the expression of apoptotic related genes, caspase-3, poly (ADP-ribose) polymerase (PARP), and BAX. Next, we show that E-cadherin, a key mediator in 3D spheroid formation, regulates ERK/Nrf2 and PI3K/Akt signaling to increase SOD2 activity. Furthermore, the transplantation of siRNA transfected spheroid MSCs showed inhibition of cartilage regeneration in the rat monosodium iodoacetate (MIA)-induced osteoarthritis (OA) model. We further hypothesize that the effect of the aggregated cells in a spheroid form will be effective in increasing cell survival and decreasing oxidative stress by enhanced SOD2 expression in 3D spheroid MSCs.

## 2. Materials and Methods

### 2.1. UCB-MSCs Culture

Neonatal UCB samples were collected from the umbilical vein after obtaining informed consent from the 5 different donors. This protocol was approved by the Institutional Review Board of MEDIPOST Co. Ltd. (MP-2014-04). The UCB samples were isolated by separating mononuclear cells (MNCs) using a Ficoll-Hypaque solution (Sigma, St. Louis, MO, USA). Separated mononuclear cells were suspended in a minimum essential medium (a-MEM; Gibco, Carlsbad, CA, USA) supplemented with 10% fetal bovine serum (FBS; Gibco, Carlsbad, CA, USA) and 50 μg/mL gentamicin (Gibco, Carlsbad, CA, USA), respectively. The cells were cultured and incubated at 37 °C in a humidified atmosphere containing 5% CO_2_. The culture medium was replaced with fresh medium twice a week, as previously described [34]. Neutralizing antibody treatment was performed to block E-cadherin expression. After trypsinization of the monolayered UCB-MSCs, as described above, they were then cultured in hanging drops that contained 20 μL of spheroid medium containing 100,000 cells/drop with E-cadherin neutralizing antibody (Sigma, St. Louis, MO, USA) or immunoglobulin G (R&D Systems, Minneapolis, MN, USA). The spheroid was incubated at 37 °C for 72 h. UCB-MSCs used in all experiments were at passage 6.

### 2.2. Spheroid Formation of UCB-MSCs

3D spheroids of UCB-MSCs were formed using the hanging drop technique, cell density of 1 × 10^4^ (10k) or 1 × 10^5^ (100k) per 20 μL to allow spheroid formation for up to 3 days (Figure S1). Spheroid cultures were maintained in DMEM/F12 medium containing 20% KnockOut serum replacement (SR, Gibco, Carlsbad, CA, USA), 50 μg/mL gentamicin, 100 mM nonessential amino acid (Invitrogen, Carlsbad, CA, USA), and 0.1 mmol/L 2-mercaptoethanol (Sigma, St. Louis, MO, USA). Drops were added to a non-tissue culture-treated petri dish by using a multichannel pipette, and phosphate-buffered saline (PBS) was added to the bottom of the dish to prevent evaporative loss. After 72 h, spheroids were collected via a pipette.

### 2.3. 2-D Electrophoresis

Parental naive and spheroid cells were solubilized with 2-dimensional electrophoresis (DE) buffer (7 M urea, 2.8 M thiourea, 40 mM Tris, 4% CHAPS, pH 8.5), sonicated using a probe sonicator, and centrifuged at 12,000× *g* rpm. 2-DE with immobilized pH gradients (IPG) using IPG strips, pH 4–7 or pH 3–10, and the IPGphor isoelectric focusing system was conducted for resolving protein extracts. The IPG strips were equilibrated in a solution containing 6 M urea, 50 mM Tris (pH 8.8), 30% glycerol, 2% sodium dodecylsulfate (SDS), and 0.5% dithiothreitol (DTT). Solution containing 4.5% iodoacetamide instead of DTT in previous solution was newly changed. Second-dimension sodium dodecylsulfate polyacrylamide gel (sodium dodecylsulfate polyacrylamide gel (SDS-PAGE), 12.5%) was used for following electrophoresis in a PROTEAN II xi 2-D cell (50 mA, Bio-Rad Laboratories, Hercules, CA, USA) system with instruction manual. Thereafter, the silver-staining was performed to visualize the all protein spots in 2-DE gels.

### 2.4. In-Gel Digestion and Mass Spectrometry Analysis

The protein species of interest were manually excised from gels and prepared for liquid chromatography-tandem mass spectrometry (LC-MS/MS). All spots were isolated from resolved gels, de-stained of silver dye using a 1:1 solution of 30 mM potassium ferricyanide and 100 mM sodium thiosulfate. Further, in-gel digestion was conducted with trypsin (10 ng/μL, Promega, Madison, WI, USA). A matrix-assisted laser desorption ionization time-of-flight (MALDI-TOF) plate containing a solution of alpha-cyano-4-hydroxycinnamic acid (Sigma-Aldrich Chemie GmbH, Taufkirchen, Germany) in 50/50 CAN/0.2% trifluoroacetic acid (TFA, Fisher Scientific GmbH, Waltham, MA, USA) was then applied onto digested-peptide sections, followed by the manufacturer’s protocol. Analysis of mass spectrometry was done with a Voyager-DE™ STR workstation (Applied Biosystems, Framingham, MA, USA). Mascot was used to search the edited peak list against the Swiss-Prot database. Protein scores (>56) were accepted as positive matches based on probability (*p* ≤ 0.05). In case of multiple hits for the same set of *m/z* values, the sequences of each peak were manually checked.

### 2.5. Reverse Transcription Polymerase Chain Reaction and RNA Interference

Total RNAs were isolated from 3D spheroids of UCB-MSCs and their adherent cells using TRIzol reagent (Invitrogen, Carlsbad, CA, USA). cDNA was prepared using the cDNA synthesis kit (Roche, Basel, Switzerland), according to the manufacturer’s instructions. UCB-MSCs were prepared and transfected with 25 nM SOD2 siRNA and scrambled-control siRNA (Dharmacon, Inc., Lafayette, CO, USA) with DharmaFECT reagent for 24 h. Transfected MSCs were then trypsinized and used to form the spheroid in the culture at 37 °C for 3 days. The primer pairs and siRNA targeting sequence are described in Table S1.

### 2.6. Western Blot Analysis

Proteins were collected from 3D spheroids of UCB-MSCs using the radioimmunoprecipitation assay buffer (RIPA), which is a lysis buffer containing protease inhibitors (Thermo Fisher Scientific, Waltham, MA, USA); proteins were then sonicated. Protein samples were separated and transferred onto nitrocellulose membranes. After blocking, the membranes were stained with primary antibodies: PARP, p-ERK, t-ERK, p-AKT and t-AKT (cell signaling, Danvers, MA, USA), BAX, SOD2, E-cadherin, PI3K and p-Nrf2 (Abcam, Cambridge, UK), caspase-3 (Santa Cruz Biotech., Dallas, TX, USA), GAPDH (AbClon, Seoul, Korea), and β-actin (Sigma), respectively. After washing, the membranes were incubated with the HRP-conjugated secondary antibody. Protein bands were visualized using an Amersham™ ECL Plus system (GE Healthcare, Chicago, IL, USA) and imaged using a ChemiDoc XRS camera (Bio-Rad Laboratories, Hercules, CA, USA). Band densities were analyzed via Image Lab software 6.0.1 (BioRad) and calculated by normalization to GAPDH or β-actin.

### 2.7. Immunofluorescent Staining

Spheroids were dissociated into single cells with 0.1% collagenase solution. Dissociated cells were washed and then seeded at a density of 4 × 10^4^ cells/2 cm^2^. Fixed and permeabilized cells were blocked, followed by staining with primary antibodies for super-oxidative dismutase (Abcam, Cambridge, UK), and then incubated with Alexa 488-conjugated secondary antibodies (BD Biosciences, San Jose, CA, USA). Stained cells were photographed using a confocal laser-scanning microscope (LSM 700, Zeiss, Oberkochen, Germany).

### 2.8. Superoxide Dismutase 2 Activity Assay

Prior to measuring activity of SOD2, mitochondrial protein extracted from spheroid cells was isolated using the mitochondria/cytosol fraction kit (Abcam, Cambridge, UK). Superoxide dismutase activity was measured using the superoxide dismutase assay kit, according to the manufacturer’s instructions (Sigma). SOD2 activity was measured in the presence of a copper/zinc superoxide dismutase inhibitor (Cu/Zn SOD). Data were normalized to protein content. Absorbance values were measured at 450 nm. Each sample be assayed in triplicates. The calculation of SOD activity (inhibition rate %) followed the manufacturer’s equation.

### 2.9. Acquisition and Treatment of Spheroid with *Hyaluronic Acid*/Synovial Fluid

All patients provided written informed consent. This protocol was approved by the Institutional Review Board of Samsung Medical Center (SMC-2012-10-078-019). Synovial fluid (SF) was withdrawn from the knee joints of patient diagnosed with grade 4 OA. SF samples were centrifuged at 300× *g* for 5 min to remove macromolecules and cell debris, the supernatants were stored at –80 °C until required for analysis. SF was diluted twice with 1% hyaluronic acid (HA), and then the cells were exposed with HA/SF (3 × 10^6^ cells/400 μL) mixture in an e-tube for 16 h.

### 2.10. Live/Dead Staining

Apoptotic and living cells in spheroids were stained with two distinct fluorescence dyes (Molecular Probes, MoBiTec, Göttingen, Germany). Spheroids were harvested and incubated for 10 min with 2 μM Calcein-AM and 4 μM ethidium homodimer-1, respectively. The spheroids were then separated into a single cell with 0.1% collagenase solution for accurate cell counting and fluorescent analysis. In order to compare pictures, fluorescence intensities were normalized using the maximum value, using Image J 1.5 software (NIH, Bethesda, MD, USA).

### 2.11. Determination of ROS Levels

The levels of intracellular ROS were determined by a 2′,7′-dichlorodihydro-fluorescein diacetate assay (DCFH-DA, Cayman). Briefly, spheroids and their adherent cells were exposed to a HA/SF (3 × 10^6^ cells/400 μL) mixture at 37 °C for 16 h. The washed cells were co-treated with 0.1% collagenase and 10 μM DCFH-DA, respectively, at 37 °C for 30 min. Then, the cells were evaluated by flow cytometry using a MACS Quant analyzer (Miltenyi Biotec, Gladbach, Germany).

### 2.12. Animal Models and Histopathological Analysis

This study was performed in accordance with the Animal Experimentation Policy of KNOTUS Co. Ltd. The protocol and procedures involving the care or use of animals in this study were reviewed and approved by the Study Facility Institutional Animal Care and Use Committee before the initiation of any such procedures (KNOTUS IACUC 16-KE-084). In brief, Sprague-Dawley male rats (6 weeks old, 200 g) were anesthetized by intra-peritoneal injection of Zoletil 50 (VIRBAC, Carros, France, 5 mg/kg) and xylazine (Rompun^®^, Bayer AG, Leverkusen, Germany, 2.5 mg/kg). A medial para-patellar arthrotomy was made, full flexion position of the right knee was then set in order to expose the intra-articular joint space. The anterior cruciate ligament (ACL) was then transected with scissors and the skin was closed. After 7 days, rats were injected with 3 mg of monosodium iodoacetate (MIA, Sigma) in 50 µL volume; a 26 G needle was inserted through the patellar ligament into the intra-articular space of the knee. After 2 weeks from the MIA injection, ten 100k spheroids (1 × 10^5^ cells) in 50 μL of 0.5% HA were injected using a 1 mL needle. After 8 weeks, the rats were euthanized using carbon dioxide. The number of rats used in this experiment is described in Table S2.

For histopathological analysis, the joints were cut 0.5 cm above and below the joint line, fixed with 10% formalin, and then decalcified with 12.5% ethylenediaminetetraacetic acid (EDTA). Then hematoxylin and eosin (H&E), Safranin O, and type II collagen stained slides were prepared after paraffin embedding and micro-sectioning. For type II collagen staining, the slides were stained with primary antibody against type II collagen (Millipore, Billerica, MA, USA), followed by detection using the DAB kit (K4006, DAKO, Carpinteria, CA, USA).

For in vivo imaging, the cells (1.0 × 10^6^ cells/mL) were re-suspended in phosphate-buffered saline. After cell labeling with Neostain 749 (NEO science, Gyeonggi, Korea) for 20 min, the cells were left to form the spheroid. Then, labeled cells were transplanted into the rat knee as described above, and the rats were observed for 8 weeks. The rats were anesthetized and placed into the in vivo imaging system chamber (FOBI Ver3.1, NEO science, Gyeonggi, Korea). Images were acquired at week 1, 2, 3, 4, 6, and 8, respectively, using fluorescence filters [Cy7]. Data analysis was performed using NEOimage for FOBI software (NEO science, Gyeonggi, Korea).

### 2.13. Statistical Analyses

Data are presented as mean ± standard deviation (SD). Statistical analysis was performed using one-way ANOVA with Fisher’s least significant difference posthoc test for multiple comparisons in GraphPad Prism version 6 software (GraphPad Software, La Jolla, CA, USA); *p*-values less than 0.05 were considered statistically significant.

## 3. Results

### 3.1. Identification of SOD2 in 3D Spheroid Form of UCB-MSCs

**Figure 1:**
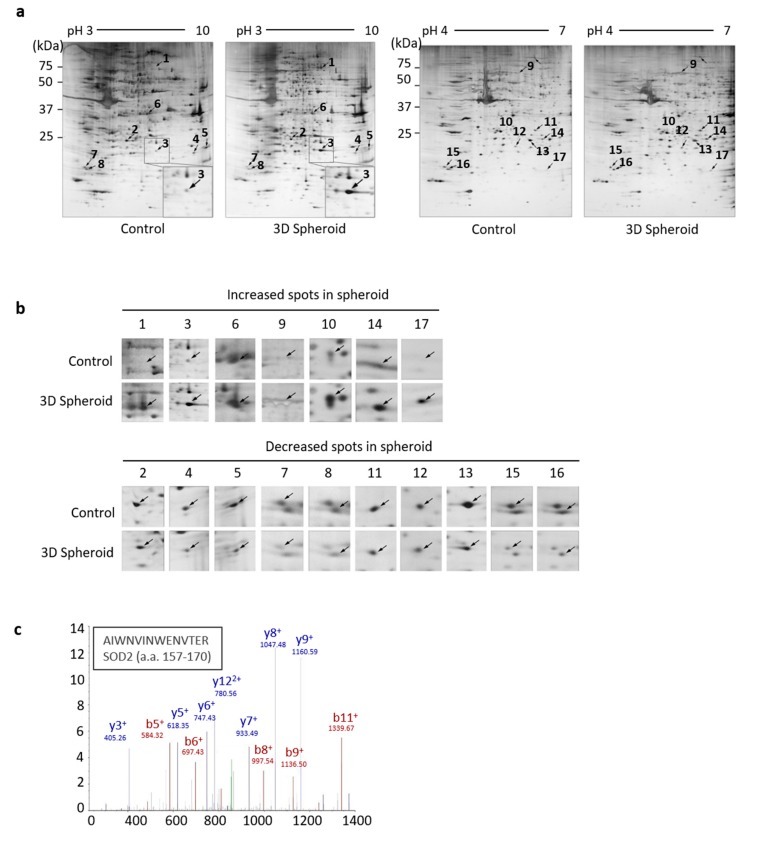
The 3D spheroid culture of mesenchymal stem cells (MSCs) caused significant changes in superoxide dismutase 2 (SOD2) gene expression. (**a**) Representative silver-stained 2-dimensional electrophoresis (DE) gels (pH 3–10, pH 4–7 isoelectric focusing (IEF), 12.5% sodium dodecylsulfate polyacrylamide gel (SDS-PAGE)) of protein extracts from 3D-spheroid MSCs were shown to determine the difference in protein expression. Identified spots are numbered with the corresponding identities of the definitive secretome proteins listed in Table 1. (**b**) Increased and decreased spots in the secretome analysis of 3D spheroid are listed. (**c**) A representative liquid chromatography-tandem mass spectrometry (LC-MS/MS) product obtained from spot 3 indicate the detection of b-ions in red and y-ions in blue. The corresponding peptide sequence was identified as SOD2 peptide 157–170. Abbreviations: a.a.—amino acids.

To better understand the aggregation of MSCs, comparative proteomic analysis was employed on 3D spheroid UCB-MSCs formed by the hanging drop technique and cultured for 3 days. The proteins extracted from the control and 3D spheroid groups were resolved by IPG 2DE. Assignments were obtained for non-redundant 2D gel spots resolved at pH 3–10 and pH 4–7 gradients, respectively, as well as acrylamide concentrations (Figure 1a). Densitometric quantification of relative spot intensities for the protein species resolved within the 2D-gel revealed a distribution in terms of the extent of protein change by silver staining. However, there were significant changes of spot intensities for protein species that were detected in gels of the 3D spheroid culture. Numbered spots correspond to changed protein species listed in Figure 1b and Table 1. Among the 17 protein species with significant changes of intensities, 7 specific spots of upregulated proteins were detected, and 10 proteins decreased in overall abundance (Figure 1b). Protein identification was stratified by iterative bio-informative screening using the Proteome Discoverer search engine (Table 1). 

Except for non-identified proteins (spot 1 and 17) who exhibited upregulation when in spheroid formation, the relative intensity of spot 3 had a high 4.14-fold change. To determine specific protein alterations between control and 3D spheroid MSCs, distinct secretome analysis was carried out by ion trap MS/MS from the 2D gel sample after in-gel trypsin digestion. The protein identity of spot 3 was confirmed by matching multiple peptide spectra, or by a single peptide with manual inspection of the MS/MS ion spectrum. The corresponding sequence was identified as SOD2 peptide 157–170. Thus, by the analysis of secretome data, this complementary protein identification demonstrated that the enhanced SOD2 expression was associated with 3D spheroid formation of MSCs.

### 3.2. The Increased Expression of SOD2 in 3D Spheroid Formation

**Figure 2:**
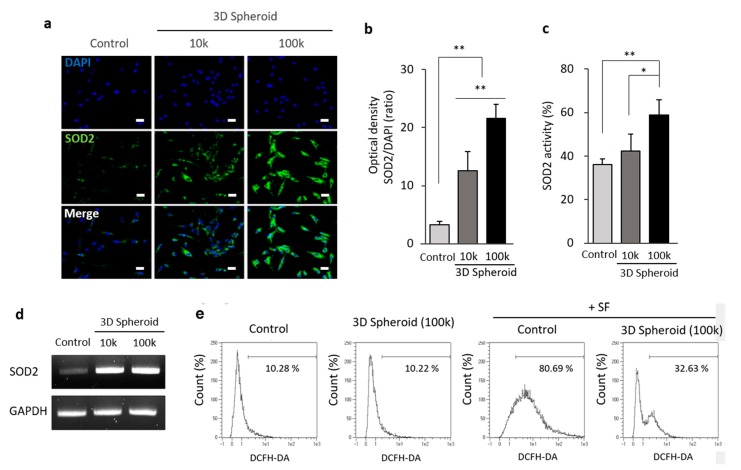
Enhanced expression of SOD2 in spheroids of UCB-MSCs. UCB-MSCs, and spheroids (3D) were cultured for 3 days under at 37 °C and 5% CO_2_ conditions. 3D spheroid MSCs were maintained in DMEM/F12 medium containing 20% serum replacement (SR) for aggregation. (**a**) Immunofluorescent staining of human SOD2 on 3D-spheroid MSCs was done after dissociation into single cells with 0.1% collagenase. The nuclei were stained by DAPI. (**b**) The optical density of SOD2 normalized to DAPI. (**c**) The activity test of SOD2 was measured after the fraction of mitochondria. (**d**) RT-PCR of SOD2 was analyzed on spheroids and monolayers in UCB-MSCs. (**e**) Intracellular reactive oxygen species (ROS, H_2_O_2_) generation was reduced by 3D spheroid formation of MSCs. SF obtained from the knee joints of osteoarthritis (OA) patients was treated. 2D and 3D spheroid MSCs (100k) were exposed to SF/HA mixture for 16 h. ROS (H_2_O_2_) was monitored by FACS analysis to observe any increase in fluorescence probe, 2′,7′-dichlorodihydro-fluorescein diacetate assay (DCFH-DA). Fluorescent intensity was determined at excitation wavelength 488 nm and emission wavelength 525 nm. Scale bars = 10 μm. Data is presented as mean ± SD, *n* = 3 per group. * *p* < 0.05. ** *p* < 0.01. Abbreviations: SOD2—superoxide dismutase; DAPI—(4′,6-diamidino-2-phenylindole); FACS—Fluorescence Activated Cell Sorting.

To verify the expression of SOD2 in the 3D spheroid of MSCs, an investigation into the immunofluorescent detection of SOD2 in spheroid was performed. There was an increase of SOD2 protein expression on a density of 10^5^ aggregated cells (100k), compared to that of 10^4^ aggregated cells (10k) in a spheroid form (Figure 2a). SOD2 increased, up to 5-fold, the proportion of MSCs on the 100k MSC spheroid form by analyzing the optical density and comparing it to the nucleus (Figure 2b). Moreover, the activity of SOD2 revealed that a significant increase was observed on the spheroid of 100k MSCs. Further, a 1.5-fold increase in the proportion of the spheroid of 100k MSCs was detected. However, no significant increase in SOD2 activity on the spheroid of 10k MSCs was measured (Figure 2c). The gene expression and protein level of SOD2 on the spheroid forms of MSCs were determined by RT-PCR (Figure 2d).

We determined the clearance of mitochondrial ROS in spheroid MSCs to further examine the function of SOD2. The levels of extracellular ROS were measured by DCFH-DA and flow cytometry. Synovial fluid (SF), obtained from the knee joints of patients who had been diagnosed with OA, was treated on MSCs for 16 h to mimic severe osteoarthritic conditions. After labeling with DCFH-DA, ROS production on control and 3D spheroid culture was assessed. The fluorescence intensity of ROS on control and 3D spheroid showed no significant difference between the control (10.28%) and the 3D (10.22%) spheroid group which had no treatment of SF. However, with the treatment of SF on MSCs, the fluorescence intensity of ROS was 80.69%. However, the ROS level in the 3D spheroid group substantially decreased to 32.63% when compared to the control group with the osteoarthritic condition (Figure 2e). Taken together, these findings indicated that spheroid MSCs in 3D cellular interactions increased the expression of SOD2, triggering the clearance of mitochondrial ROS.

### 3.3. SOD2 in 3D Spheroid MSCs Related to Apoptosis

**Figure 3:**
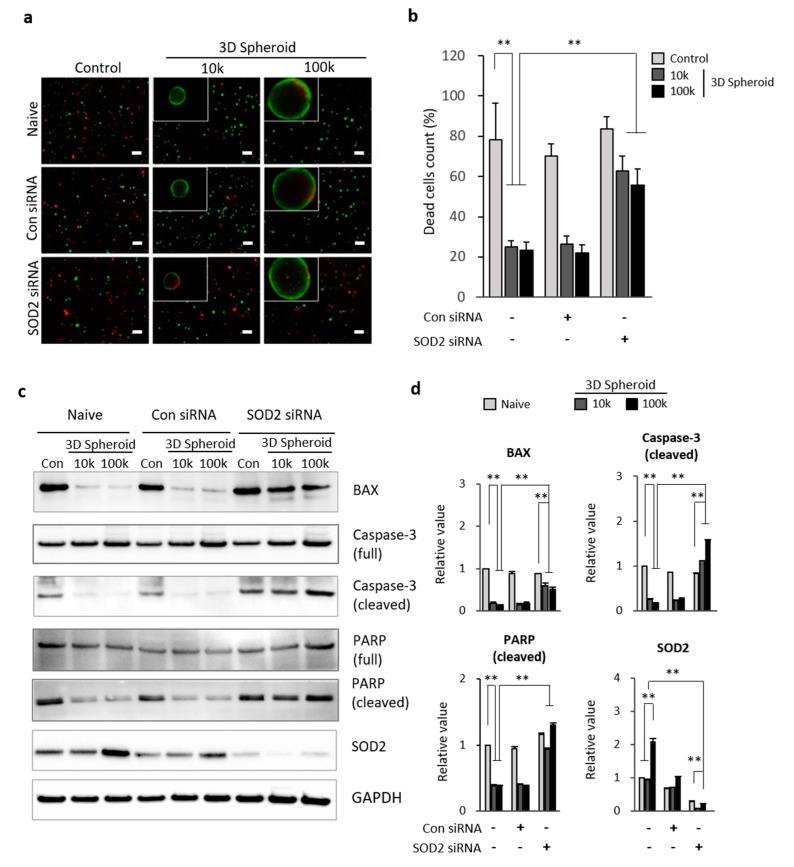
SOD2 knockdown induces apoptosis of 3D spheroid MSCs. 3D spheroid (10k and 100k) MSCs were transfected with 25 nM scramble-siRNA (Con siRNA) and SOD2 siRNA, respectively, for 24 h. The spheroid (10k and 100k) of transfected MSCs was collected via pipette after 72 h incubation in synovial fluid to mimic the OA in in vitro. (**a**) 3D spheroid MSCs (10k and 100k) embedded in HA/SF mixture (SF:HA = 1:1) overnight were stained with live/dead staining after 0.1% collagenase dissociation. (**b**) Live (green, 2 μM calcein-AM) and dead (red, 4 μM ethidium homodimer-1) cells were counted for quantitative analysis of dead cells. (**c**) Western blot analysis was done for apoptosis-related proteins of 3D spheroid MSCs in SF after SOD2 siRNA transfection for 24 h. GAPDH was used as a loading control. (**d**) Relative value of protein expression, BAX, Caspase-3 (cleaved), and PARP (cleaved), and SOD2 was normalized to GAPDH. Scale bars = 10 μm. Data are presented as mean ± SD, *n* = 3 per group. ** *p* < 0.01. Abbreviations: SOD2—superoxide dismutase 2; BAX—BCL2 associated X; PARP—poly (ADP-ribose) polymerase; SF—synovial fluid.

To determine the role of SOD2 in 3D spheroid MSCs, and as a result, elicit protective properties against cell death, we suppressed the SOD2 gene expression by transfecting MSCs with 25 nM SOD2 siRNAs for 24 h. After transfection, the spheroid formed for 72 h was incubated in SF overnight. Live and apoptotic MSCs were stained with calcein-AM (green) and ethidium homodimer (red), respectively, following dissociation with 0.1% collagenase. Apoptotic cells in cell densities of 10k and 100k with 3D spheroid form had decreased, compared to the control in naive condition. However, the SOD2 knockdown MSCs did not have a significant difference of apoptotic cells between the control and spheroidal shape groups. The increase in percentages of apoptotic cells was detected in 3D spheroid form of SOD2 siRNA transfected MSCs compared to the control and control siRNA transfected groups (Figure 3a,b). The level of apoptotic-related proteins on 3D spheroid MSCs transfected with SOD2 siRNA was analyzed by way of Western blot analysis. BAX, caspase-3 (cleaved), and PARP (cleaved) protein levels were decreased on 3D spheroid MSCs (10k and 100k) on both naive and control siRNA groups compared to control. Additionally, SOD2 protein levels were decreased on both control and 3D spheroid MSCs with SOD2 siRNA transfection. The 3D spheroid formation of the SOD2 siRNA transfected MSCs showed a significant increase of caspase-3 (cleaved) compared to control. The degree of decrease in BAX and PARP (cleaved) proteins was slightly reduced with SOD2 siRNA transfection in the spheroid form of MSCs (Figure 3c,d). Overall, SOD2 expression increased in the spheroid form of MSCs and promoted the suppression of apoptotic related genes, BAX, caspase-3 (cleaved), and PARP (cleaved). As a result, a decrease in the levels of apoptosis in cells was observed.

### 3.4. Mechanism Related to SOD2 Mediated by E-Cadherin in 3D Spheroid Form of MSCs

**Figure 4:**
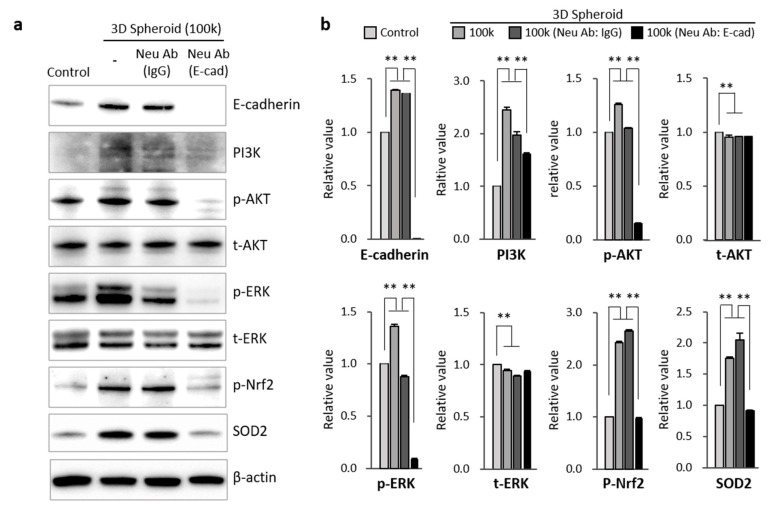
Mechanism of SOD2 mediated by E-cadherin of 3D spheroid MSCs. (**a**) MSCs were treated with E-cadherin neutralizing antibody or IgG isotype control antibody. The 3D spheroid MSCs were formed after 72 h incubation. Protein extracts from 3D spheroid and 2D MSCs were stained with immuno-blot antibodies, E-cadherin, PI3K, p-AKT, t-AKT, p-ERK, t-ERK, p-Nrf2, and SOD2. (**b**) Relative value of each protein level was normalized to β-actin. Data are presented as mean ± SD, *n* = 3 per group. ** *p* < 0.01. Abbreviations: SOD2—superoxide dismutase 2.

According to previous studies, the formation of 3D spheroids is dependent on the calcium-dependent cell adhesion molecule, E-cadherin. Furthermore, it has also been identified as a critical component of stem cells ability to resist against oxidative stress [29,35,36]. To clarify the relationship of E-cadherin and the high expression of SOD2 seen in 3D spheroid formation, a neutralizing antibody (400 μg/mL) of E-cadherin was used over a period of 72 h while the spheroid was forming. The restoration of cell-to-cell interaction via 3D spheroid formation improved the expression of E-cadherin. We confirmed that E-cadherin was necessary for the 3D spheroid formation in MSCs. E-cadherin neutralization inhibited 3D spheroid formation (Figure S2). Through Western blot analysis, there was a significant increase in E-cadherin and SOD2 expression in the 3D spheroid formation of MSCs (Figure 4a). However, the treatment of neutralizing antibody bound to E-cadherin directly interfered with its function, resulting in no expression of E-cadherin on 3D spheroid MSCs. Also, the downstream signaling proteins of E-cadherin, PI3K, p-AKT, p-ERK, and p-Nrf2, were increased with 3D spheroid formation of MSCs. The neutralization of E-cadherin on 3D spheroid MSCs led to the significant inhibition of PI3K, p-AKT, p-ERK, or the activation of p-Nrf2. The protein expressions of t-AKT and t-ERK showed no significant change with 3D spheroid formation. Interestingly, the SOD2 expression of 3D spheroid MSCs was downregulated with the blocking of E-cadherin (Figure 4b). As a result, we confirmed that 3D spheroid formation increased E-cadherin and SOD2 expression, which caused upregulation of the PI3K/pAkt/pNrf2 and pERK/pNrf2 signaling pathways.

### 3.5. Enhanced Cartilage Repair in Spheroid-Injected OA Animal Model

**Figure 5:**
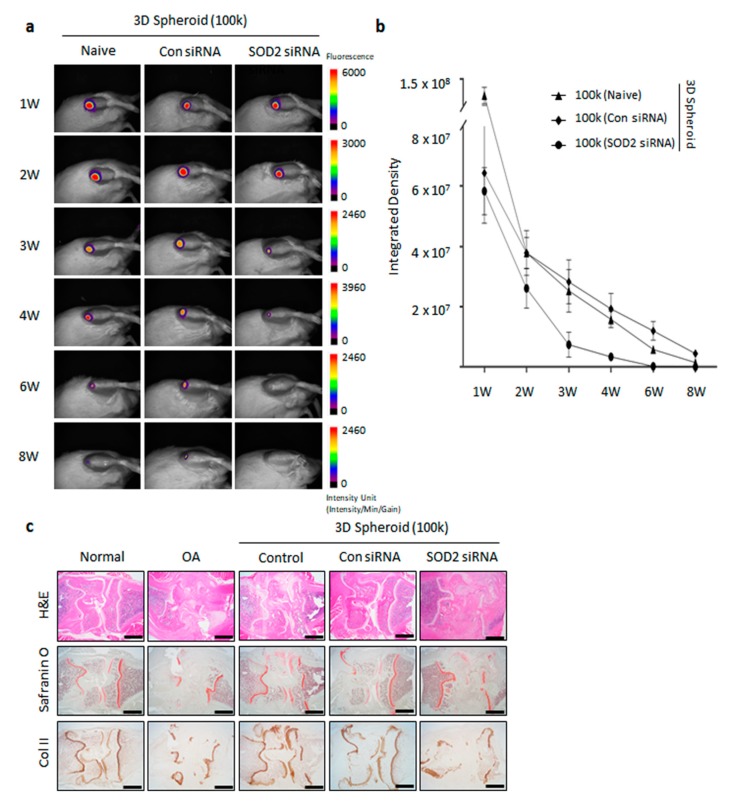
In vivo imaging and histopathological analysis in an monosodium iodoacetate (MIA)-induced osteoarthritis (OA) rat model. (**a**) In vivo imaging. 3D spheroid formed by MSCs labeled with Neostain 749 were transplanted into the MIA-induced OA rat knee for an 8-week observation period. Anesthetized rats were scanned through the in vivo imaging system (FOBI) chamber. The images were taken at week 1, 2, 3, 4, 6 and 8, respectively, with fluorescence filters [Cy7]. SOD2 siRNA transfected MSCs were used to form the 3D spheroid (100k). (**b**) Integrated density was analyzed using the NEOimage program at week 1, 2, 3, 4, 6 and 8, respectively. (**c**) SD rats were injected with 3 mg of monosodium iodoacetate (MIA) to induce the osteoarthritis in knee. The knee joints from MIA-induced OA rats treated with 3D spheroid of SOD2 siRNA transfected MSCs (100k) with 0.5% HA. The knee joints were stained with hematoxylin and eosin (H&E), safranin O fast green, and type II collagen (Col II) antibody. Scale bars = 250 μm. *n* = 3 for 2D, Control, SOD2 siRNA group. *n* = 2 for Con siRNA group. *n* = 3 for Naive, SOD2 siRNA group. *n* = 2 for Con siRNA group. Abbreviations: SOD2—superoxide dismutase 2; DCFH-DA—dichloro-dihydro-fluorescein diacetate; OA—osteoarthritis; HA—hyaluronic acid; MIA—monosodium iodoacetate.

Having observed the association of enhanced SOD2 expression in 3D spheroid MSCs, we next explored the influence of SOD2 in regeneration of cartilage in an MIA-induced OA rat model. Thus, we first examined the ability of spheroid MSCs to reside in the knee of our rat models. 3D spheroid MSCs labeled with Neostain 749 were transfected for an 8-week observation period (Figure 5a and Figure S3). The integrated density of 3D spheroid MSCs was similar among the control, Con siRNA, and SOD2 siRNA groups, respectively. The leftover of transfected 3D spheroid MSCs with con siRNA and SOD2 siRNA was observed for 8 weeks. The majority of transplanted cells had disappeared after 8 weeks (Figure 5b).

To determine whether SOD2 expression, enhanced by 3D spheroid MSCs, has an effect on the regeneration of cartilage, we implanted 3D spheroid MSCs transfected with SOD2 siRNAs. Histological analysis revealed that the chondrogenic response was well generated in the control and Con siRNA transfected spheroid MSCs groups. Both groups showed that newly generated cartilage was evident. However, the SOD2 siRNA transfected spheroid MSC group had not regenerated as an incomplete structure of the knee remained. The generation of structural cartilage by MSCs was not clear in the SOD2 siRNA transfected group. Non-cartilaginous tissue filled up the osteochondral defect at 8 weeks in spheroid SOD2 siRNA transfected MSCs. The deposition of sGAG and proteoglycan in regenerated cartilage on the defect site was markedly augmented by spheroid MSCs and confirmed by safranin O staining. Immunohistochemical staining for collagen type II antibody and mature hyaline cartilage was observed at 8 weeks and showed significant staining for type II collagen. Overall, in vivo imaging and immunohistochemical analysis highlighted that the SOD2 expression on 3D spheroid MSCs induces optimum cartilage regeneration.

## 4. Discussion

In this study, we investigated a new approach that has the potential to optimize the characteristics of MSCs cultured using a 3D spheroid culture system. Tightly adhered and condensed cells in 3D spheroid MSCs maintain and mimic intercellular functions, showing the ability to recapitulate characteristics of tissue in vivo. We found that the knockdown of SOD2, which is highly expressed in 3D spheroid MSCs, resulted in an increase in the apoptosis of MSCs. Furthermore, this study evaluated that cell adhesion molecule, E-cadherin, is enhanced in 3D spheroid MSCs and as a result, it regulates ERK/Nrf2 and PI3K/Akt signaling, followed by an increase of SOD2 expression. Additionally, we confirmed that the knockdown of SOD2 affected the regeneration of cartilage in an MIA-induced OA rat model. Taken together, our results demonstrated that our 3D spheroid culture system is efficient for maximizing the efficacy of MSCs by increasing overall cell survival, decreasing oxidation stress, and increase chondrogenic activity by triggering SOD2 expression.

There were several strategies for optimizing the functions of MSCs, including genetic modifications or manipulation of culture conditions [37]. Spheroid culture resembles the features of tissues and serves as a model system to study cell-cell interactions in a 3D environment. For instance, the aggregation of MSCs in 3D spheroid form can significantly enhance the differentiation capacity, anti-inflammatory properties, proangiogenic ability, and therapeutic potency [19,29,38,39]. Even though the altered gene expression profiles, which are related to hypoxia, angiogenesis, inflammation, stress response, proliferation, migration, and redox signaling, in MSC spheroids were performed with microarray analysis, the molecular mechanisms, however, are largely unknown [18,40]. Additionally, maximizing therapeutic efficacy and safety still remains a great challenge for MSC-based therapies [41]. Therefore, spheroid formation of cell culture has the potential to improve therapeutic efficacy.

From the secretome analysis and mass spectrum of 3D spheroid MSCs, we identified the specific increased protein as SOD2. We listed the significantly increased and decreased proteins with identification on Table 1 (Figure 1b). In our results, the expression of SOD2 in spheroid MSCs was significantly increased compared to the control group (Figure 1). The 10^5^ aggregated cells (100k) had higher expression and activity of SOD2 than 10^4^ aggregated cells (10k) by immunofluorescence and activity analysis (Figure 2a–c). Also, there was an increase in SOD2 gene expression observed in 3D spheroid MSCs (Figure 2d). Intracellular reactive oxygen species levels, such as ROS and H_2_O_2_, increased after the introduction of SF taken from the knee joints of OA patients. However, in spheroid MSCs, ROS levels were decreased. This reduction of ROS is thought to be due to the increase of SOD2 expression seen in our spheroid MSCs (Figure 2e).

SOD2 is known to limit the detrimental effects of ROS and is crucial for various cellular processes, including cell growth, apoptosis, cell adhesion, and immune responses [42]. SOD2 transforms toxic superoxide into hydrogen peroxide, allowing the clearance of mitochondrial ROS and protection against cell death [43]. Previous research has shown that the transplantation of SOD2-overexpressed UCB-MSCs through the tail veins of rats results in a decrease in inflammatory cytokines, tumor necrosis factor (TNF-α), interleukin-8 (IL-8), and ICAM-1 in the bronchoalveolar lavage fluid (BALF) of paraquat-induced lung injured rat. Also, the overexpression of SOD2 changed the oxidative stress index in lung tissues and attenuated lung injury [44]. Overall, SOD2 has a role in preventing apoptosis due to its protective role against oxidative stress as an endogenous scavenger of oxygen free radicals, ionizing radiation, and inflammatory cytokines, [32,45,46]. Various reports suggest that ROS allows NF-κB translocation to the nucleus, activating the transcription genes for inflammatory factors, cytokines, and chemokines [42,46]. Considering the relationship between oxidative stress and apoptosis, we then expected to observe the increase in apoptosis-related genes, BAX, caspase-3, and PARP, in spheroid form with SOD2 knockdown MSCs. Caspase-3 are crucial mediators of the initiation and execution of apoptotic processes directly involved in programmed cell death [47]. In our study, cleaved caspase-3, which reflects the progression of apoptosis of 3D spheroid formation, was significantly decreased. However, the knockdown of SOD2 in spheroid MSCs increased the expression of cleaved caspase-3. Similarly, cleaved PARP and BAX, hallmarks of apoptosis, were significantly increased with the knockdown of SOD2 in spheroid MSCs (Figure 3). Thus, we postulate that the anti-apoptotic effects in 3D spheroid MSCs compared to single cells can be due to highly expressed SOD2 in spheroid MSCs, with anti-apoptotic properties due to the protective role of SOD2 against oxidative stress.

Although the current study may not be able to conclusively prove the mechanisms by which the spheroid consistently outperformed the attached cells, we show convincingly that aggregated MSCs allows better extracellular and intercellular communication. Cell-to-cell interaction on aggregated MSCs is the main factor that increases SOD2 expression in spheroid cultures, as seen in our results. Cadherins, which are mediators of specific cell–cell adhesion in a calcium-dependent manner, have critical roles in morphogenesis and cell adhesion in a variety of organs [48]. Based on the change of SOD2 expression seen in our 3D spheroid culture, we assumed that E-cadherin could be a key mediator in the formation of the spheroidal MSCs. To assess the role of E-cadherin in intercellular adhesion of spheroid formation, we blocked E-cadherin. Neutralization of the extracellular domain of E-cadherin using a neutralizing antibody inhibited the formation of MSCs. According to previous research, E-cadherin was significantly increased during the process of spheroid formation, which is in accordance with our results (Figure 4). No previous reports have described the direct relationship between E-cadherin and SOD2 on MSCs. The reports related to the relationship between E-cadherin and MSCs are limited to epithelial-mesenchymal transition (EMT) of stem cells. SOD2 is involved in the earlier stage of transition of E-cadherin-to-N-cadherin during EMT of stem cells [33]. Given the requirement of mitochondrial superoxide at the onset of EMT, SOD2 may inhibit EMT in epithelial cells by suppressing mitochondrial ROS. As suggested in epithelial cells, E-cadherin homophilic interactions on aggregated MSCs activated the regulatory mechanism of cell survival via the AKT and ERK signaling pathways [29,48,49]. This activation of the AKT/ERK signal pathway correlated with our results that the blockade of E-cadherin using a neutralizing antibody inhibited the signaling pathways of PI3K/pAKT/pNrf2 and ERK/pNrf2, leading to activation of proliferative and paracrine properties of spheroid MSCs.

OA, caused by aging and incurs injury to joints, is a slow progressive degenerative disease characterized by degradation of the matrix and cell death, which ultimately results in joint pain and stiffness [50,51]. Previously, mitochondrial dysfunctions have been associated with the pathological onset of osteoarthritis. An increased level of oxidative stress is observed in OA models [52]. Superoxide is a ROS, mainly generated during mitochondrial respiration and metabolized by SOD2 in mitochondria. An increase of ROS could potentially contribute to the onset of senescence, triggered in OA [53,54]. From the analysis of the degenerative cartilage in osteoarthritis patients, a pathological relationship between SOD2 downregulation and cartilage degeneration has been observed [55,56,57]. However, the protective and regenerative effects of SOD2 related to osteoarthritis have not been fully elucidated. Previous investigations suggest that intra-articular injection of a permeable antioxidant effectively suppresses mitochondrial superoxide generation and cartilage degeneration in SOD2 knockout mice [55]. In the present study, we found that the spheroid of SOD2 siRNA transfected MSCs had no significant effect on cartilage regeneration in MIA-induced OA rats. However, histological analysis of the knee joints clearly revealed that cartilage destruction was significantly attenuated after the intra-articular injection of the spheroid MSCs. Together, we suggest that SOD2 is a critical factor in improving the regeneration of cartilage in OA patients (Figure 5c).

**Figure 6:**
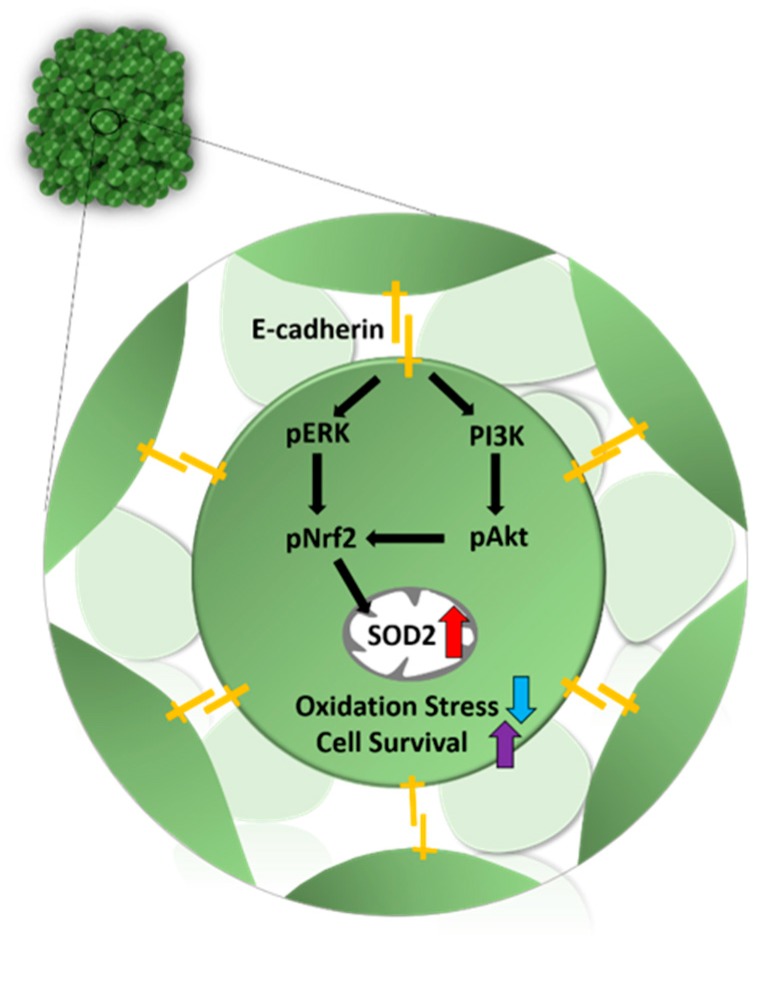
Schematic mechanism of SOD2 mediated by E-cadherin. The E-cadherin on aggregated cells mediates cell-cell adhesion. Activated E-cadherin from interaction with other cells triggered the pERK and PI3K pathways, followed by the activation of pAkt and pNrf2. Consequently, pNrf2 regulates the expression of SOD2 in mitochondria to decrease oxidative stress and increase cell survival. Symbol: E-cadherin (yellow), Increased action (red and purple), and Decreased action (blue).

Nrf2 is an important endogenous regulator of antioxidative stress and plays a pivotal role in oxidative-stress management through antioxidation and anti-inflammatory effects. Nrf2 signaling is related to antioxidative and damage-control processes [58,59,60]. Previously, SOD2 overexpression in UCB-MSCs was reported to induce Nrf2 expression, revealed in the lung of injured rats. There were protective effects against the lung injury through the activation of Nrf2 signaling [44]. Our experimental data showed that pNrf2 was increased with the spheroid formation of MSCs. However, the knockdown of SOD2 in spheroid MSCs had shown the downregulation of Nrf2 expression. To our knowledge, our results demonstrate that increased expression of E-cadherin by spheroid formation of MSCs regulated the ERK/Nrf2 and PI3K/pAkt/pNrf2 signaling pathways to accelerate SOD activity, which is directly related to cell survival through antioxidative action (Figure 6).

## 5. Conclusions

This is the first study to analyze the effects of spheroid MSCs, which exhibit high expression of SOD2. Downregulation of oxidative stress, apoptosis, and degeneration of cartilage in knee joints of OA has been highlighted in our study. Herein, we propose a new therapeutic approach, 3D spheroid formation of MSCs, for improving the regeneration process in the course of OA. The increase in SOD2 in the spheroid form of MSCs is a particularly relevant finding relative to the improvement of therapeutic potency by enhancing cell survival and suppressing the oxidative stress seen in OA.

## Figures and Tables

**Table 1:** Mass spectrometry analysis by 3D spheroids identified by 2-D gel electrophoresis.

Spot No.	Name	MW	PI	Score (MOWSE)	Cov %	Swiss-Prot Assession No	±Fold/ (Control)
1	No identification						4.81
2	Guanylyl cyclase-activating protein 1	22,920	4.3	7341	29.4	P43080	0.61 (–1.64)
3	Superoxide dismutase [Mn], mitochondrial	24,722	8.3	181	71.0	P04179	4.14
4	Transgelin-2	22,392	8.4	17,935	36.2	P37802	0.64 (–1.56)
5	No identification						0.62 (–1.61)
6	Aldose reductase	35,854	6.5	16,298	24.4	P15121	1.41
7	Myosin regulatory light chain 12A	19,794	4.7	10,303	40.1	P19105	0.27 (–3.70)
8	Myosin regulatory light chain 12B	19,779	4.7	25,759	40.1	O14950	0.41 (–2.44)
9	Serum albumin (Bovin)	69,294	5.8	5,100,000	19.4	P02769	2.88
10	Cathepsin D	44,553	6.1	1,930,000	34.5	P07339	2.95
11	Glutathione S-transferase omega-1	27,566	6.2	41,480	25.7	P78417	0.66 (–1.52)
12	Ribosomal RNA small subunit methyltransferase NEP1	26,720	9.3	2202	27.9	Q92979	0.62 (–1.61)
13	Guanylyl cyclase-activating protein 1	22,920	4.3	7341	29.4	P43080	0.61 (–1.64)
14	Triosephosphate isomerase	26,670	6.4	7037	30.5	P60174	1.54
15	Myosin regulatory light chain 12A	19,794	4.7	11,511	34.9	P19105	0.27 (–3.70)
16	Myosin regulatory light chain 12B	19,779	4.7	25,759	40.1	O14950	0.45 (–2.22)
17	No identification						5.60

MW—Molecular Weight; PI—Isoelectric point; MOWSE—Molecular Weight Search; Cov—covariance; NEP—nucleolar essential protein 1.

## Supplementary Materials

The following are available online at https://www.mdpi.com/2076-3921/9/1/66/s1, Figure S1: Schematic illustration of our experimental scheme. Control group was kept with single cells. 3D spheroid groups consisted of aggregated 10k and 100k MSCs. Figure S2: E-cadherin Neutralizing antibody condition on spheroid formation. (a) After forming the spheroid, the various concentrations of E-cadherin neutralizing antibody, 40, 200, 400, and 1000 μg/mL, respectively, were treated on the spheroid formed of UCB-MSCs for periods of 7, 22, 30, 54, and 75 h, respectively. (b) After forming the spheroid for 72 h, the 400 μg/mL IgG and E-cadherin neutralizing antibody were treated for 7, 24, 48, and 72h. (c) The protein levels of E-cadherin and SOD2 of UCB-MSCs were analyzed with western blot on 3D spheroid MSCs. (d) The gene expressions of E-cadherin and SOD2 were determined with western blot analysis after treatment of neutralizing antibody on 3D spheroid MSCs. Figure S3: Characteristics of UCB-MSCs transfected with 25 nM SOD2 siRNA transfection. (a) Effects of SOD2 knockdown on UCB-derived MSCs. MSCs were transfected with 25 nM scramble siRNA (Con siRNA) or SOD2 siRNA (SOD2 siRNA) for 24 h. SOD2 levels were measured by qPCR. Expression levels were normalized to GAPDH, with the expression levels in the Control defined as 1. (b,c) UCB-MSCs transfected with SOD2 siRNA labeled with Neostain 749 for 20 min. After labeling, the spheroid was formed, and the intensity of fluorescence was analyzed. Error bars represent the means ± SD, n = 3 per group. Table S1: Primer and siRNA set. Table S2: Experimental design in vivo.

## Abbreviations

UCB-MSC	Umbilical cord blood-derived MSCs
2D	Two-dimensional
3D	Three-dimensional
RGD	Arginylglycylaspartic acid
ACSC	Adipose-derived stem cells
BMSCs	Bone marrow-derived stem cells
WJCs	Wharton’s jelly cells
SOD2	Superoxide dismutase 2
MIA	Monosodium iodoacetate
OA	Osteoarthritis
MNCs	Mononuclear cells
SR	Serum replacement
PBS	Phosphate-buffered saline
DE	Dimensional electrophoresis
SDS-PAGE	Sodium dodecylsulfate polyacrylamide gel
LC-MS/MS	Liquid chromatography-tandem mass spectrometry
MALDI-TOF	Matrix assisted laser desorption ionization time-of-flight
SF	Synovial fluid
HA	Hyaluronic acid
BALF	Bronchoalveolar lavage fluid
EMT	Epithelial-mesenchymal transition
PI3K	Phosphoinositide 3-kinase
Akt	Alpha serine/threonine-protein kinase
Nrf	Nuclear respiratory factor
ERK	Extracellular signal-regulated kinases
