# Supplementary material for: Up-Regulation of Superoxide Dismutase 2 in 3D Spheroid Formation Promotes Therapeutic Potency of Human Umbilical Cord Blood-Derived Mesenchymal Stem Cells
Source: Antioxidants (Basel). 2020 Jan 11;9(1):66. doi: 10.3390/antiox9010066 (PMC7023074; doi:10.3390/antiox9010066)
Supplement: Supplementary file 1 [file antioxidants-09-00066-s001.pdf]

**Supplementary Materials:**

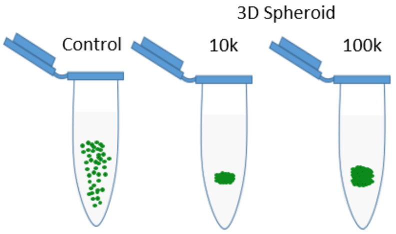


**Figure S1**. Schematic illustration of our experimental scheme. Control group was kept with single cells. 3D spheroid groups consisted of aggregated 10k and 100k MSCs.

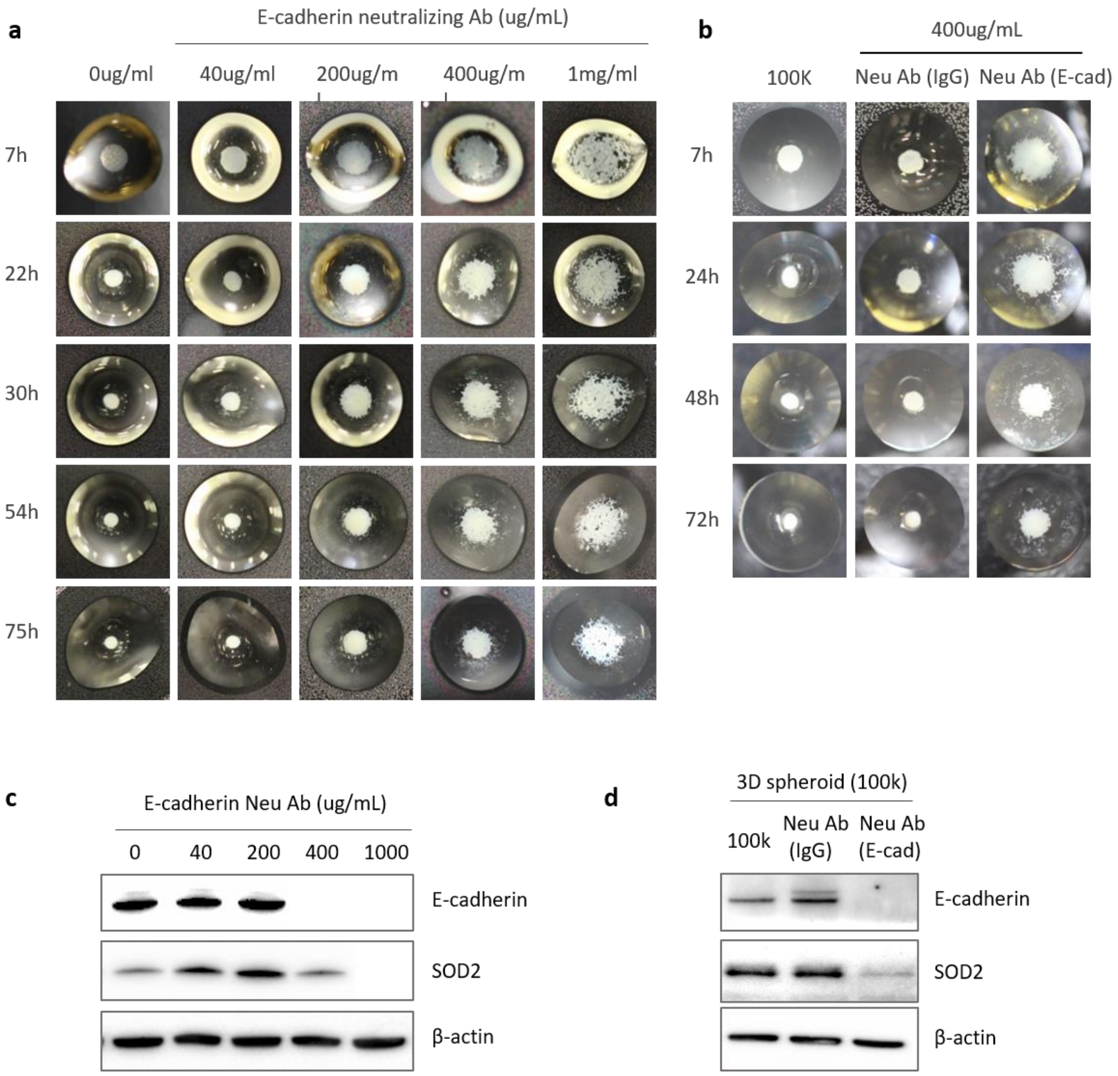


**Figure S2**. E-cadherin Neutralizing antibody condition on spheroid formation. (**a**) After forming the spheroid, the various concentrations of E-cadherin neutralizing antibody, 40, 200, 400, and 1000 μg/mL, respectively, were treated on the spheroid formed of UCB-MSCs for periods of 7, 22, 30, 54, and 75 h, respectively. (**b**) After forming the spheroid for 72 h, the 400 μg/mL IgG and E-cadherin neutralizing antibody were treated for 7, 24, 48, and 72 h. (**c**) The protein levels of E-cadherin and SOD2 of UCB-MSCs were analyzed with western blot on 3D spheroid MSCs. (**d**) The gene expressions of E-cadherin and SOD2 were determined with western blot analysis after treatment of neutralizing antibody on 3D spheroid MSCs.

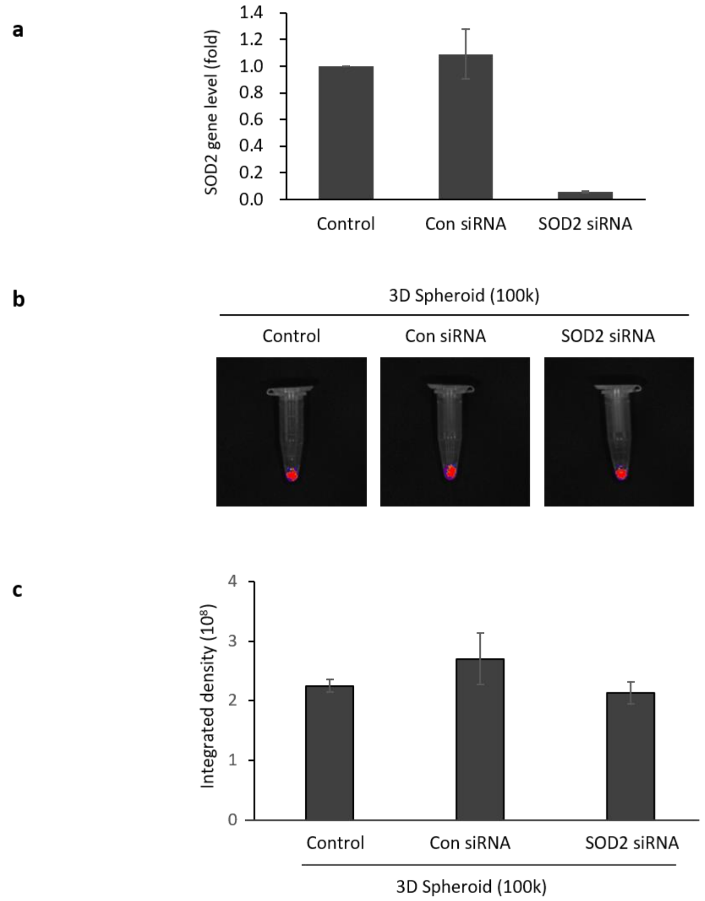


**Figure S3**. Characteristics of UCB-MSCs transfected with 25 nM SOD2 siRNA transfection. (**a**) Effects of SOD2 knockdown on UCB-derived MSCs. MSCs were transfected with 25 nM scramble siRNA (Con siRNA) or SOD2 siRNA (SOD2 siRNA) for 24 h. SOD2 levels were measured by qPCR. Expression levels were normalized to GAPDH, with the expression levels in the Control defined as 1. (**b**,**c**) UCB-MSCs transfected with SOD2 siRNA labeled with Neostain 749 for 20 min. After labeling, the spheroid was formed, and the intensity of fluorescence was analyzed. Error bars represent the means ± SD, $n = 3$ per group.

**Table S1.** Primer and siRNA set

| Gene | Primer Sequence (5′ -> 3′) |
| --- | --- |
| GAPDH | CTTCTTTTGCGTCGCCAGCCGA<br>TGGCCAGGGGTGCTAAGCAGT |
| SOD2 | AAACCTCAGCCCTAACGGTG<br>TGGCCAGGGGTGCTAAGCAGT |
| Scramble siRNA | UGGUUUACAUGUCGACUAA<br>UGGUUUACAUGUUGUGUGA<br>UGGUUUACAUGUUUUCUGA<br>UGGUUUACAUGUUUUCCUA |
| Human SOD2 siRNA | AAAGAUACAUGGCUUGCAA<br>AAGUAAACCACGAUCGUUA<br>GUAAUCAACUGGGAGAAUG<br>CAACAGGCCUUAUUCCACU |

**Table S2.** Experimental design in vivo

| Group | Bio-imaging | Histology |
| --- | --- | --- |
| Normal | | 5 |
| OA | | 5 |
| OA + 3D Spheroid (100k, Control) | 3 | 8 |
| OA + 3D Spheroid (100k, Con siRNA) | 2 | 7 |
| OA + 3D Spheroid (100k, SOD2 siRNA) | 3 | 8 |
